# The genome sequence of the large white, *Pieris brassicae* (Linnaeus, 1758)

**DOI:** 10.12688/wellcomeopenres.17274.1

**Published:** 2021-10-12

**Authors:** Konrad Lohse, Alexander Mackintosh, Darwin Tree of Life Barcoding collective, Wellcome Sanger Institute Tree of Life programme, Wellcome Sanger Institute Scientific Operations: DNA Pipelines collective, Tree of Life Core Informatics collective, Darwin Tree of Life Consortium

**Affiliations:** 1 Institute of Evolutionary Biology, University of Edinburgh, Edinburgh, UK

**Keywords:** Pieris brassicae, large white, genome sequence, chromosomal

## Abstract

We present a genome assembly from an individual female *Pieris brassicae* (the large white; Arthropoda; Insecta; Lepidoptera; Pieridae). The genome sequence is 292 megabases in span. The majority of the assembly is scaffolded into 16 chromosomal pseudomolecules, with the W and Z sex chromosome assembled. Gene annotation of this assembly on Ensembl has identified 12,229 protein coding genes.

## Species taxonomy

Eukaryota; Metazoa; Ecdysozoa; Arthropoda; Hexapoda; Insecta; Pterygota; Neoptera; Endopterygota; Lepidoptera; Glossata; Ditrysia; Papilionoidea; Pieridae; Pierinae; Pieris; *Pieris brassicae* (Linnaeus, 1758) (NCBI:txid7116).

## Introduction

The large white, *Pieris brassicae*, is a Palearctic butterfly species that is common in Europe, North Africa, and Asia. *P. brassicae* larvae typically feed on Brassicaceae species, including cultivated species with agricultural importance such as *Brassica oleracea*. It has been unintentionally introduced to New Zealand, Chile, and South Africa, although it was later eradicated from New Zealand in 2016 ( Phillips *et al.*, 2020). It overwinters as a pupa and is multivoltine. While *P. brassicae* has been listed as Least Concern in the IUCN Red List (Europe), the Madeiran large white, *P. wollastoni* (previously considered a subspecies of *P. brassicae*), has not been observed since 1986 and is possibly extinct ( IUCN, 2009). *P. brassicae* has 15 pairs of chromosomes with the female being heterogametic ( Bigger, 1975). This karyotype is unusual *,* as species in the genus *Pieris* typically possess between 24 and 28 pairs of chromosomes ( Robinson, 1971). Its genome size has been estimated with flow cytometry at approximately 260 Mb ( Mackintosh *et al.*, 2019).

## Genome sequence report

**Figure 1.**
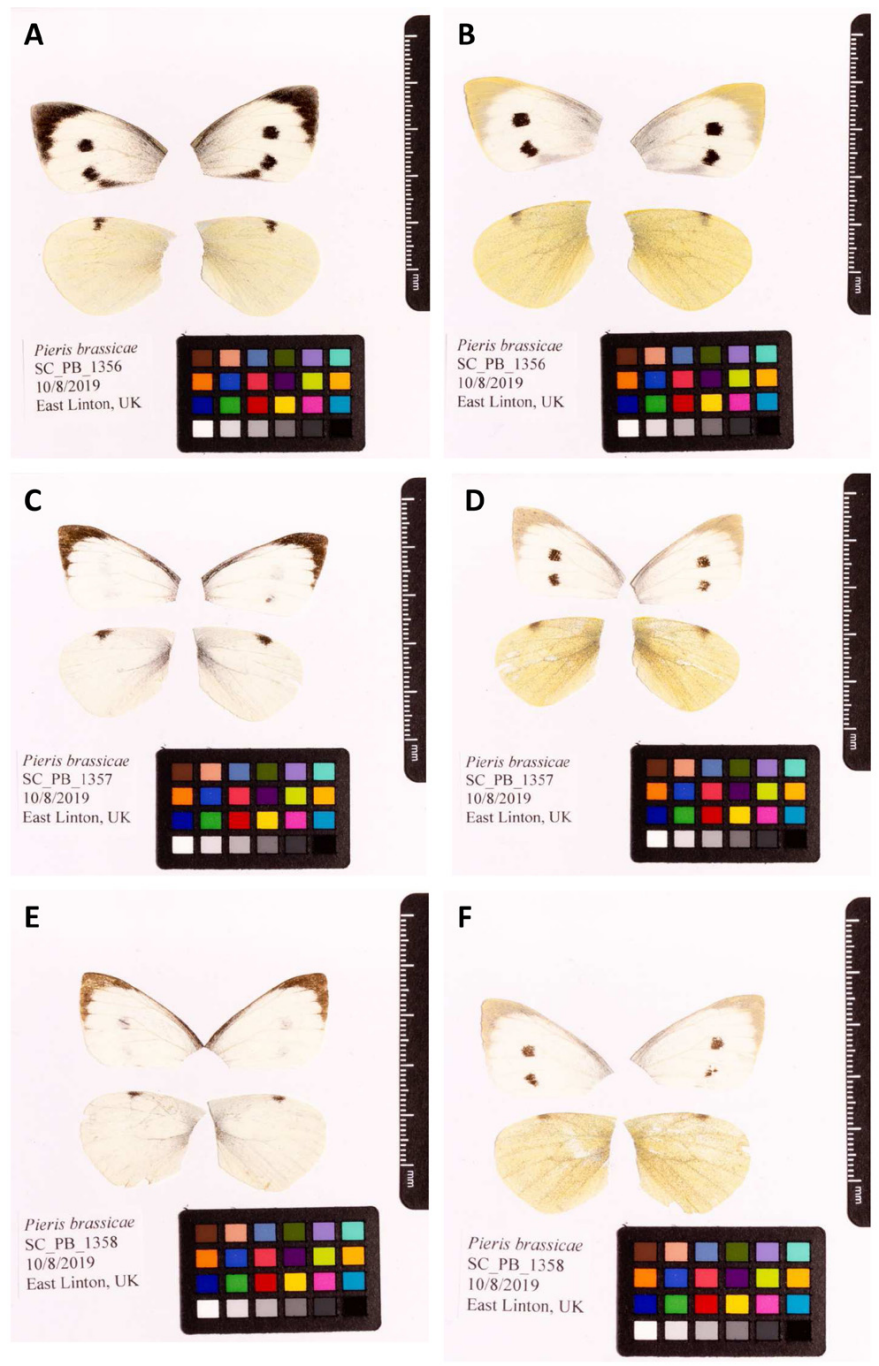
Fore and hind wings of *Pieris brassicae* specimens from which the genome was sequenced. (A) Dorsal surface view of wings from specimen SC_PB_1356 ( **ilPieBrab1**) from East Linton, used to generate Pacific Biosciences and 10X genomics data. (B) Ventral surface view of wings from specimen SC_PB_1356 (ilPieBrab1) from East Linton, used to generate Pacific Biosciences and 10X genomics data. (C) Dorsal surface view of wings from specimen SC_PB_1357 (ilPieBrab2) from East Linton, used to generate RNASeq data. (B) Ventral surface view of wings from specimen SC_PB_1357 (ilPieBrab2) from East Linton, used to generate RNASeq data. (A) Dorsal surface view of wings from specimen SC_PB_1358 (ilPieBrab3) from East Linton, used to generate Hi-C data. (B) Ventral surface view of wings from specimen SC_PB_1358 (ilPieBrab3) from East Linton, used to generate Hi-C data.

The genome was sequenced from a single female *P. brassicae* (ilPieBrab1) collected from East Linton, Scotland (latitude 55.977161, longitude -2.667545); Hi-C data were generated from a male *P. brassicae* (ilPieBrab3) collected from the same location ( Figure 1). A total of 92-fold coverage in Pacific Biosciences single-molecule long reads (N50 14 kb) and 138-fold coverage in 10X Genomics read clouds were generated. Primary assembly contigs were scaffolded with chromosome conformation Hi-C data, which was obtained from a different individual (ilPieBrab3). Manual assembly curation corrected 25 missing joins, reducing the scaffold number by 5.87%, and increasing the scaffold N50 by 1.21%. 

**Figure 2.**
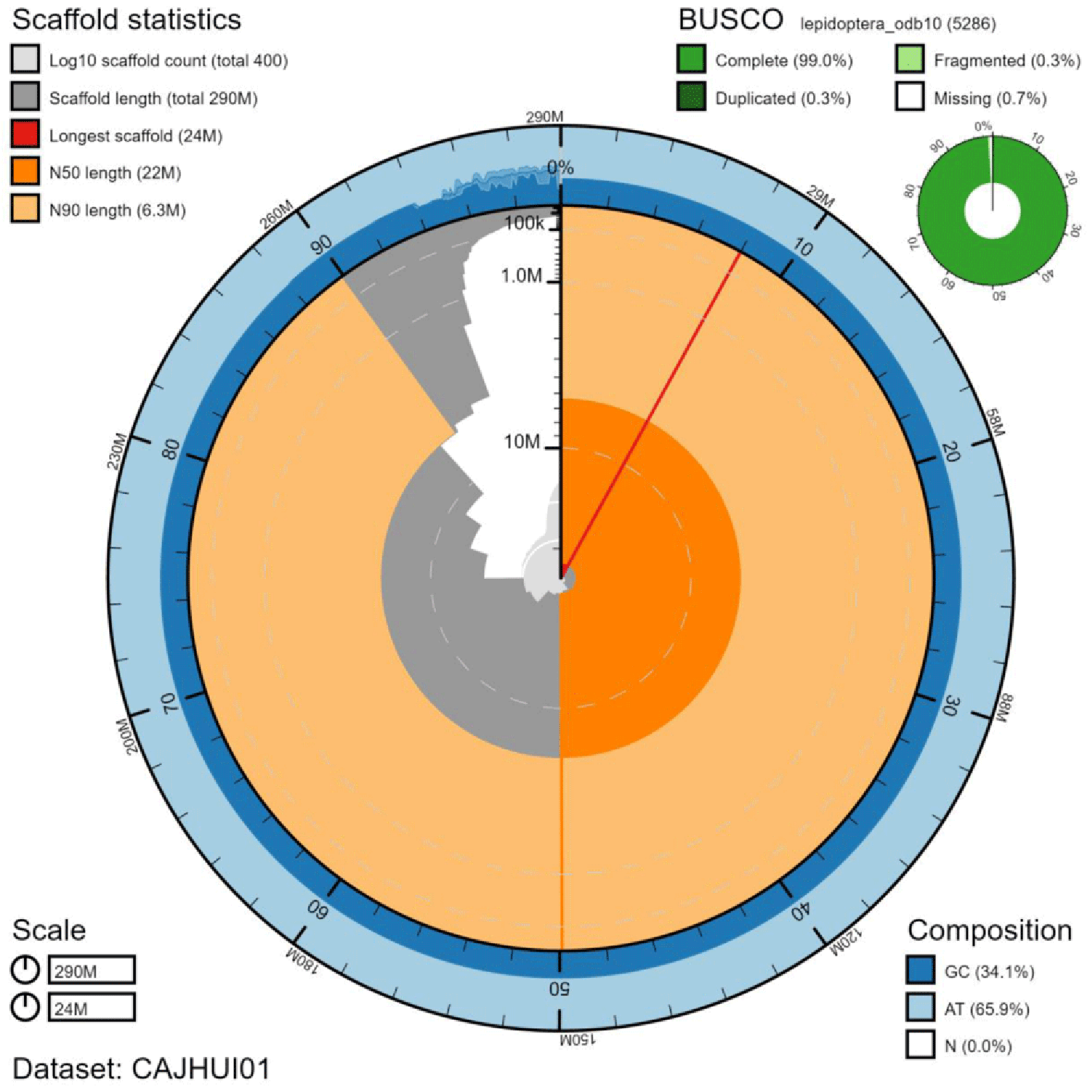
Genome assembly of *Pieris brassicae*, ilPieBrab1.1: metrics. The BlobToolKit Snailplot shows N50 metrics and BUSCO gene completeness. The main plot is divided into 1,000 size-ordered bins around the circumference with each bin representing 0.1% of the 292,341,157 bp assembly. The distribution of scaffold lengths is shown in dark grey with the plot radius scaled to the longest chromosome present in the assembly (23,587,467 bp, shown in red). Orange and pale-orange arcs show the N50 and N90 chromosome lengths (21,549,451 and 6,322,985 bp), respectively. The pale grey spiral shows the cumulative scaffold count on a log scale with white scale lines showing successive orders of magnitude. The blue and pale-blue area around the outside of the plot shows the distribution of GC, AT and N percentages in the same bins as the inner plot. A summary of complete, fragmented, duplicated and missing BUSCO genes in the lepidoptera_odb10 set is shown in the top right. An interactive version of this figure is available at https://blobtoolkit.genomehubs.org/view/ilPieBrab1.1/dataset/CAJHUI01/snail.

The final assembly has a total length of 292 Mb in 402 sequence scaffolds with a scaffold N50 of 22 Mb ( Table 1). Of the assembly sequence, 95.46% was assigned to 16 chromosomal-level scaffolds, representing 14 autosomes (numbered by sequence length), and the W and Z sex chromosome ( Figure 2– Figure 5; Table 2). The W chromosome is fragmented as the assembly was scaffolded to an individual of a different sex (ilPieBrab3). The assembly has a BUSCO ( Simão *et al.*, 2015) completeness of 99.0% using the lepidoptera_odb10 reference set. While not fully phased, the assembly deposited is of one haplotype. Contigs corresponding to the second haplotype have also been deposited.

**Figure 3.**
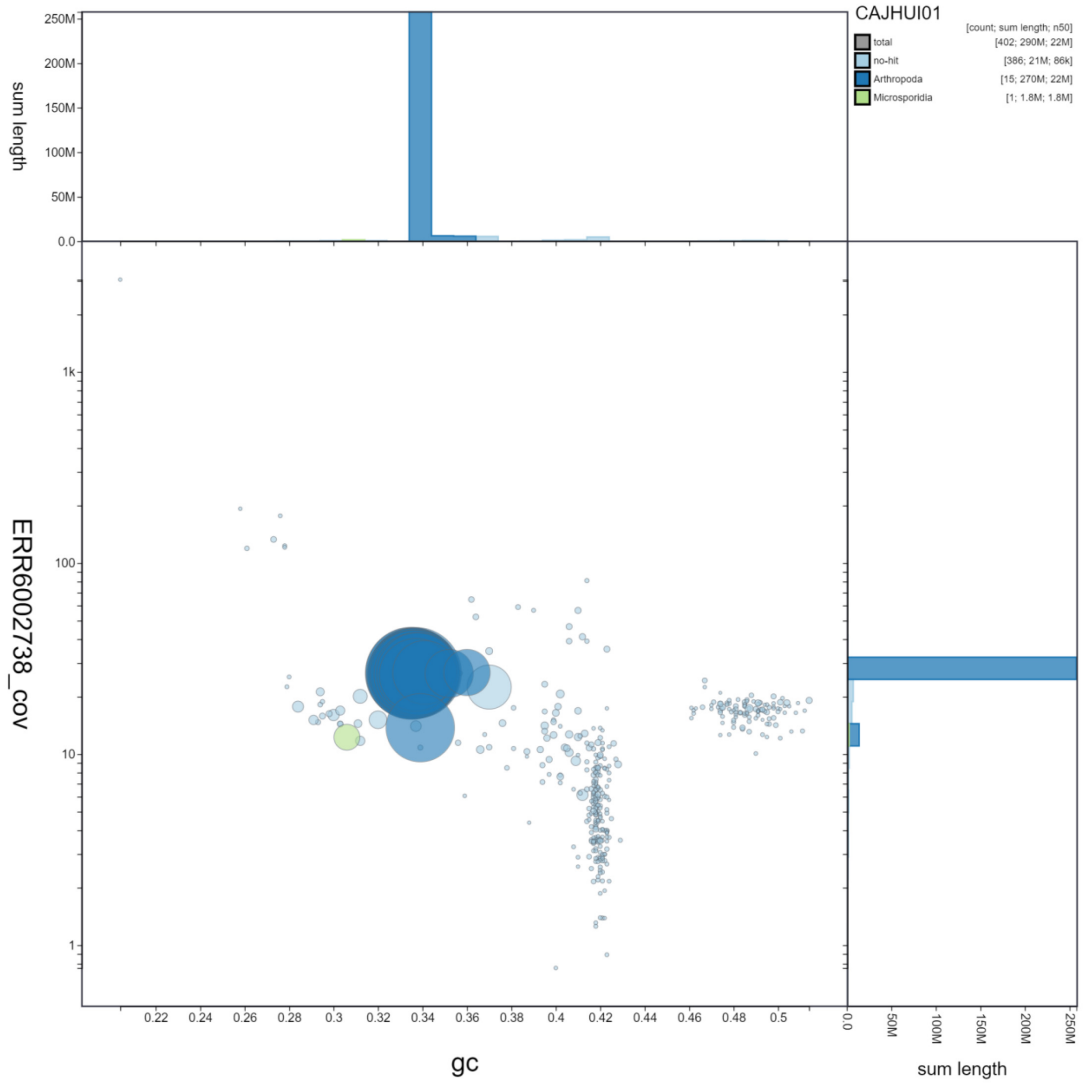
Genome assembly of *Pieris brassicae*, ilPieBrab1.1: GC coverage. BlobToolKit GC-coverage plot. Scaffolds are coloured by phylum. Circles are sized in proportion to scaffold length. Histograms show the distribution of scaffold length sum along each axis. An interactive version of this figure is available at https://blobtoolkit.genomehubs.org/view/ilPieBrab1.1/dataset/CAJHUI01/blob.

**Figure 4.**
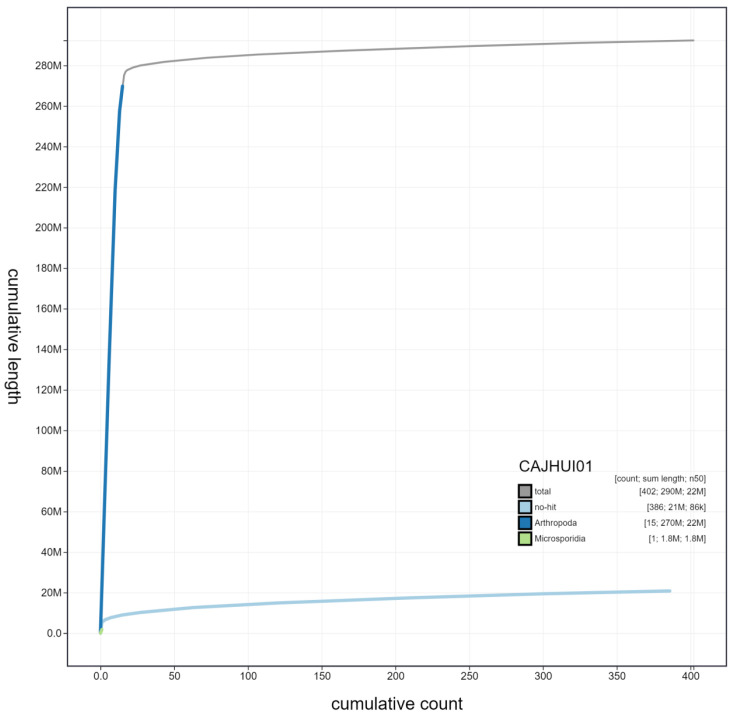
Genome assembly of *Pieris brassicae*, ilPieBrab1.1: cumulative sequence. BlobToolKit cumulative sequence plot. The grey line shows cumulative length for all scaffolds. Coloured lines show cumulative lengths of scaffolds assigned to each phylum using the buscogenes taxrule. An interactive version of this figure is available at https://blobtoolkit.genomehubs.org/view/ilPieBrab1.1/dataset/CAJHUI01/cumulative.

BlobToolKit blob and cumulative sequence plots show that the W chromosome has regions with microsporidian origin ( Figure 3, Figure 4). However, these regions in the read sets are short, do not match across the rest of the scaffold and do not contain any contigs with microsporidian ribosomal subunits. This indicates that this feature is unlikely to be contamination and is more likely to be the result of integration of microsporidian sequence into the genome.

**Table 1.** Genome data for *Pieris brassicae*, ilPieBrab1.1.

*Project accession data*	
Assembly identifier	ilPieBrab1.1
Species	*Pieris brassicae*
Specimen	ilPieBrab1 (genome assembly), ilPieBrab2 (RNA-Seq), ilPieBrab3 (Hi-C)
NCBI taxonomy ID	NCBI:txid7116
BioProject	PRJEB41887
BioSample ID	SAMEA7532735
Isolate information	Female, whole organism (ilPieBrab1)
	Males, whole organisms (ilPieBrab2, ilPieBrab3)
*Raw data accessions*	
PacificBiosciences SEQUEL II	ERR6594497
10X Genomics Illumina	ERR6002738-ERR6002741
Hi-C Illumina	ERR6002742
Illumina PolyA RNA-Seq	ERR6594497
*Genome assembly*	
Assembly accession	GCA_905147105.1
*Accession of alternate* *haplotype*	GCA_905147085.1
Span (Mb)	292
Number of contigs	431
Contig N50 length (Mb)	21
Number of scaffolds	402
Scaffold N50 length (Mb)	22
Longest scaffold (Mb)	24
BUSCO * genome score	C:99.0%[S:98.7%,D:0.3%],F:0.3%, M:0.7%,n:5286
*Gene annotation*	
Number of protein coding genes	12,229
Average coding sequence length (bp)	1,736
Average number of exons per transcript	10
Average exon size (bp)	327
Average intron size (bp)	1687

*BUSCO scores based on the lepidoptera_odb10 BUSCO set using v5.1.2. C= complete [S= single copy, D=duplicated], F=fragmented, M=missing, n=number of orthologues in comparison. A full set of BUSCO scores is available at https://blobtoolkit.genomehubs.org/view/ilPieBrab1.1/dataset/CAJHUI01/busco.

**Figure 5.**
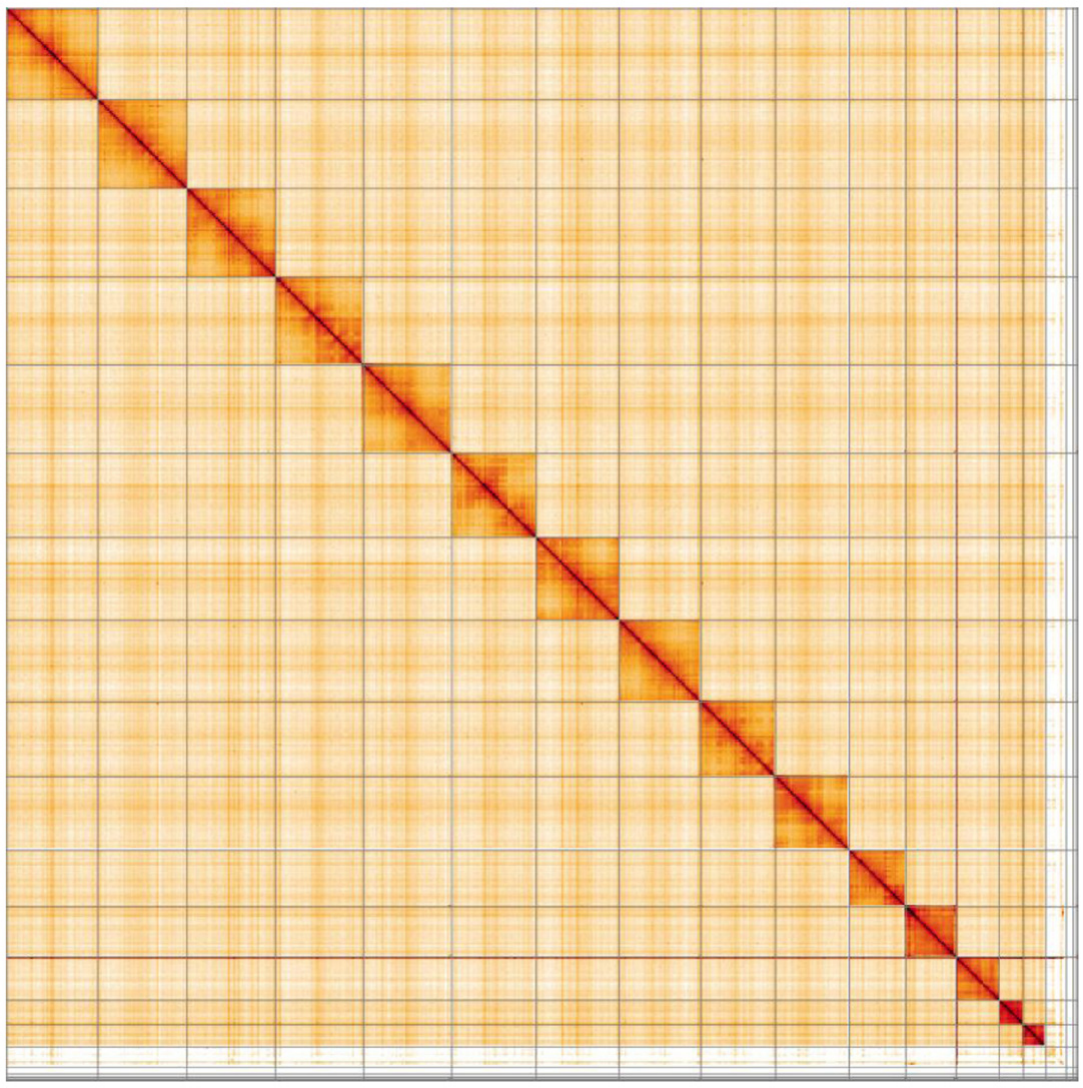
Genome assembly of *Pieris brassicae*, ilPieBrab1.1: Hi-C contact map. Hi-C contact map of the ilPieBrab1.1 assembly, visualised in HiGlass. Chromosomes are shown in order of size from left to right and top to bottom.

**Table 2.** Chromosomal pseudomolecules in the genome assembly of *Pieris brassicae*, ilPieBrab1.1.

INSDC accession	Chromosome	Size (Mb)	GC%
LR989932.1	1	23.59	33.5
LR989933.1	2	23.11	33.5
LR989934.1	3	23.07	33.7
LR989935.1	4	22.87	33.6
LR989936.1	5	22.82	33.6
LR989937.1	6	21.88	33.5
LR989938.1	7	21.55	33.7
LR989939.1	8	21.29	33.6
LR989940.1	9	19.44	33.7
LR989941.1	10	19.01	33.8
LR989942.1	11	14.79	33.7
LR989944.1	12	11.23	34.1
LR989945.1	13	6.32	35.2
LR989946.1	14	5.84	36
LR989947.1	W	5.29	37
LR989943.1	Z	12.97	33.9
LR989948.1	MT	0.02	20.7
-	Unplaced	17.25	39.1

## Gene annotation

The Ensembl gene annotation system ( Aken *et al.*, 2016) was used to generate annotation for the *Pieris brassicae* assembly (GCA_905147105.1, see https://rapid.ensembl.org/Pieris_brassicae_GCA_905147105.1/; Table 1). The annotation was created primarily through alignment of transcriptomic data to the genome, with gap filling via protein-to-genome alignments of a select set of proteins from UniProt ( UniProt Consortium, 2019) and OrthoDB ( Kriventseva *et al.*, 2008). Prediction tools, CPC2 ( Kang *et al.*, 2017) and RNAsamba ( Camargo *et al.*, 2020), were used to aid determination of protein coding genes.

## Methods

### Sample acquisition and nucleic acid extraction

A female (ilPieBrab1) and two male (ilPieBrab2, ilPieBrab3) *P. brassicae* ( Figure 1) were collected from East Linton, Scotland (latitude 55.977161, longitude -2.667545) using a net by Konrad Lohse, University of Edinburgh, who also identified the samples. The samples were snap-frozen in liquid nitrogen from live.

DNA was extracted from the whole organism of ilPieBrab1 at the Wellcome Sanger Institute (WSI) Scientific Operations core from the whole organism using the Qiagen MagAttract HMW DNA kit, according to the manufacturer’s instructions. RNA (also from the whole organism) was extracted from ilPieBrab2 in the Tree of Life Laboratory at the WSI using TRIzol, according to the manufacturer’s instructions. RNA was then eluted in 50 μl RNAse-free water and its concentration RNA assessed using a Nanodrop spectrophotometer and Qubit Fluorometer using the Qubit RNA Broad-Range (BR) Assay kit. Analysis of the integrity of the RNA was done using Agilent RNA 6000 Pico Kit and Eukaryotic Total RNA assay.

### Sequencing

Pacific Biosciences HiFi circular consensus and 10X Genomics read cloud DNA sequencing libraries were constructed according to the manufacturers’ instructions. Poly(A) RNA-Seq libraries were constructed using the NEB Ultra II RNA Library Prep kit. DNA and RNA sequencing was performed by the Scientific Operations core at the WSI on Pacific Biosciences SEQUEL II (HiFi), Illumina HiSeq X (10X) and Illumina HiSeq 4000 (RNA-Seq) instruments. Hi-C data were generated from the whole organism of ilPieBrab3 using the Qiagen EpiTect Hi-C kit and sequenced on HiSeq X.

### Genome assembly

Assembly was carried out with HiCanu ( Nurk *et al.*, 2020). Haplotypic duplication was identified and removed with purge_dups ( Guan *et al.*, 2020). One round of polishing was performed by aligning 10X Genomics read data to the assembly with longranger align, calling variants with freebayes ( Garrison & Marth, 2012). The assembly was then scaffolded with Hi-C data ( Rao *et al.*, 2014) using SALSA2 ( Ghurye *et al.*, 2019). The assembly was checked for contamination and corrected using the gEVAL system ( Chow *et al.*, 2016) as described previously ( Howe *et al.*, 2021). Manual curation was performed using gEVAL, HiGlass ( Kerpedjiev *et al.*, 2018) and Pretext. The genome was analysed and BUSCO scores generated within the BlobToolKit environment ( Challis *et al.*, 2020). Table 3 contains a list of all software tool versions used, where appropriate.

**Table 3.** Software tools used.

Software tool	Version	Source
HiCanu	1.0	Nurk *et al.,* 2020
purge_dups	1.2.3	Guan *et al.,* 2020
SALSA2	2.2	Ghurye *et al.,* 2019
longranger align	2.2.2	https://support.10xgenomics.com/ genome-exome/software/pipelines/latest/ advanced/other-pipelines
freebayes	1.3.1-17-gaa2ace8	Garrison & Marth, 2012
gEVAL	N/A	Chow *et al.,* 2016
PretextView	0.1.x	https://github.com/wtsi-hpag/PretextView
HiGlass	1.11.6	Kerpedjiev *et al.,* 2018
BlobToolKit	2.6.2	Challis *et al.,* 2020

### Ethical/compliance issues

The materials that have contributed to this genome note were supplied by a Tree of Life collaborator. The WSI employs a process whereby due diligence is carried out proportionate to the nature of the materials themselves, and the circumstances under which they have been/are to be collected and provided for use. The purpose of this is to address and mitigate any potential legal and/or ethical implications of receipt and use of the materials as part of the research project, and to ensure that in doing so we align with best practice wherever possible.

The overarching areas of consideration are:

- Ethical review of provenance and sourcing of the material;
- Legality of collection, transfer and use (national and international).

Each transfer of samples is undertaken according to a Research Collaboration Agreement or Material Transfer Agreement entered into by the Tree of Life collaborator, Genome Research Limited (operating as the Wellcome Sanger Institute) and in some circumstances other Tree of Life collaborators.

## Data availability

European Nucleotide Archive: Pieris brassicae (large white). Accession number PRJEB42142; https://identifiers.org/ena.embl/PRJEB42142.

The genome sequence is released openly for reuse. The *P. brassicae* genome sequencing initiative is part of the Darwin Tree of Life (DToL) project. All raw sequence data and the assembly have been deposited in INSDC databases. Raw data and assembly accession identifiers are reported in Table 1.

## References

[ref-1] Aken BL Ayling S Barrell D : The Ensembl Gene Annotation System. *Database (Oxford).* 2016;2016:baw093. 10.1093/database/baw093 27337980 PMC4919035

[ref-2] Bigger TRL : Karyotypes of Some *Lepidoptera* Chromosomes and Changes in Their Holokinetic Organisation as Revealed by New Cytological Techniques. *CYTOLOGIA.* 1975;40(3–4):713–726. 10.1508/cytologia.40.713

[ref-3] Camargo AP Sourkov V Pereira GAG : RNAsamba: Neural Network-Based Assessment of the Protein-Coding Potential of RNA Sequences. *NAR Genom Bioinform.* 2020;2(1):lqz024. 10.1093/nargab/lqz024 33575571 PMC7671399

[ref-4] Challis R Richards E Rajan J : BlobToolKit - Interactive Quality Assessment of Genome Assemblies. *G3 (Bethesda).* 2020;10(4):1361–74. 10.1534/g3.119.400908 32071071 PMC7144090

[ref-5] Chow W Brugger K Caccamo M : gEVAL - a web-based browser for evaluating genome assemblies. *Bioinformatics.* 2016;32(16):2508–10. 10.1093/bioinformatics/btw159 27153597 PMC4978925

[ref-6] Garrison E Marth G : Haplotype-Based Variant Detection from Short-Read Sequencing.arXiv: 1207.3907.2012. Reference Source

[ref-7] Ghurye J Rhie A Walenz BP : Integrating Hi-C Links with Assembly Graphs for Chromosome-Scale Assembly. *PLoS Comput Biol.* 2019;15(8):e1007273. 10.1371/journal.pcbi.1007273 31433799 PMC6719893

[ref-8] Guan D McCarthy SA Wood J : Identifying and Removing Haplotypic Duplication in Primary Genome Assemblies. *Bioinformatics.* 2020;36(9):2896–98. 10.1093/bioinformatics/btaa025 31971576 PMC7203741

[ref-9] Howe K Chow W Collins J : Significantly Improving the Quality of Genome Assemblies through Curation. *GigaScience.* 2021;10(1):giaa153. 10.1093/gigascience/giaa153 33420778 PMC7794651

[ref-10] IUCN: Pieris Wollastoni.Van Swaay, C., Wynhoff, I., Verovnik, R., Wiemers, M., López Munguira, M., Maes, D., Sasic, M., Verstrael, T., Warren, M. & Settele, J. *IUCN Red List of Threatened Species.*IUCN,2009. 10.2305/iucn.uk.2010-1.rlts.t39483a10240995.en

[ref-11] Kang YJ Yang DC Kong L : CPC2: A Fast and Accurate Coding Potential Calculator Based on Sequence Intrinsic Features. *Nucleic Acids Res.* 2017;45(W1):W12–16. 10.1093/nar/gkx428 28521017 PMC5793834

[ref-12] Kerpedjiev P Abdennur N Lekschas F : HiGlass: Web-Based Visual Exploration and Analysis of Genome Interaction Maps. *Genome Biol.* 2018;19(1):125. 10.1186/s13059-018-1486-1 30143029 PMC6109259

[ref-13] Kriventseva EV Rahman N Espinosa O : OrthoDB: The Hierarchical Catalog of Eukaryotic Orthologs. *Nucleic Acids Res.* 2008;36(Database issue):D271–75. 10.1093/nar/gkm845 17947323 PMC2238902

[ref-14] Mackintosh A Laetsch DR Hayward A : The Determinants of Genetic Diversity in Butterflies. *Nat Commun.* 2019;10(1):3466. 10.1038/s41467-019-11308-4 31371715 PMC6672018

[ref-15] Nurk S Walenz BP Rhie A : HiCanu: Accurate Assembly of Segmental Duplications, Satellites, and Allelic Variants from High-Fidelity Long Reads. *Genome Res.* 2020;30(9):1291–1305. 10.1101/gr.263566.120 32801147 PMC7545148

[ref-16] Phillips CB Brown K Green C : Eradicating the Large White Butterfly from New Zealand Eliminates a Threat to Endemic Brassicaceae. *PLoS One.* 2020;15(8):e0236791. 10.1371/journal.pone.0236791 32760094 PMC7410255

[ref-17] Rao SS Huntley MH Durand NC : A 3D Map of the Human Genome at Kilobase Resolution Reveals Principles of Chromatin Looping. *Cell.* 2014;159(7):1665–80. 10.1016/j.cell.2014.11.021 25497547 PMC5635824

[ref-18] Robinson R : Lepidoptera Genetics.1971. 10.1017/S0016672300011976

[ref-19] Simão FA Waterhouse RM Ioannidis P : BUSCO: Assessing Genome Assembly and Annotation Completeness with Single-Copy Orthologs. *Bioinformatics.* 2015;31(19):3210–12. 10.1093/bioinformatics/btv351 26059717

[ref-20] UniProt Consortium: UniProt: A Worldwide Hub of Protein Knowledge. *Nucleic Acids Res.* 2019;47(D1):D506–15. 10.1093/nar/gky1049 30395287 PMC6323992

