# Supplementary material for: The genome sequence of the large white, Pieris brassicae (Linnaeus, 1758)
Source: Wellcome Open Res. 2021 Oct 12;6:262. [Version 1] doi: 10.12688/wellcomeopenres.17274.1 (PMC9608253; doi:10.12688/wellcomeopenres.17274.1)
Supplement: Peer review history

# Peer review: Reviewer response for version 1

**DOI:** 10.21956/wellcomeopenres.19097.r50214

**Published:** 2022-06-21

**Authors:** Christopher Wheat

**Affiliations:** 1 Department of Zoology, Faculty of Science, Stockholm University, Stockholm, Sweden

This is a standard genome report for a new species, quickly covering the minimal aspects of the species ecology. The vast majority of genomic methods and results are reported clearly and the work is good.

 I have only three concerns. First, with regards to the polishing, it is not clear how the polishing was actually carried out (only the mapping and SNP identification are described). Details of how 10X data went through PCR duplication removal, as well as barcode removal, and ultimately how the polishing was actually performed, is never reported. Second, it is not clear how "Manual assembly curation corrected" the genome. An additional sentence here could easily fix this. Third, BUSCO reports only stating "completeness" are really quite useless. Unless people report complete single copy, the level of duplication in the BUSCO result is not known. This needs to be corrected, and more details added to the relevant figure.

Are sufficient details of methods and materials provided to allow replication by others?

Partly

Is the rationale for creating the dataset(s) clearly described?

Yes

Are the datasets clearly presented in a useable and accessible format?

Yes

Are the protocols appropriate and is the work technically sound?

Yes

Reviewer Expertise:

Butterfly genomics

I confirm that I have read this submission and believe that I have an appropriate level of expertise to confirm that it is of an acceptable scientific standard.

# Peer review: Reviewer response for version 1

**DOI:** 10.21956/wellcomeopenres.19097.r46391

**Published:** 2021-10-25

**Authors:** Will Nash

**Affiliations:** 1 The Earlham Institute, Norwich, UK

This article describes the assembly and annotation of the genome of *Pieris brassicae*, the Large White butterfly. Three individuals were used to generate the data contributing to the assembly and annotation: the first contributed high molecular weight DNA which was used for PacBio Hifi and 10X sequencing, the second contributed RNA-seq which was used in the annotation process, the third was used to generate Hi-C chromosome conformation data. 

 The rationale for this project is made clear in a well written introduction which guides the reader to the status of the agricultural importance of the species, as well as its status as an introduced species in several countries. 

 The protocols described are exemplary and in line with contemporary studies. The sequencing strategy is appropriate for an invertebrate species of the size of *P. brassicae*. The bioinformatic pipeline that is used to assemble the Hifi reads, purge haplotigs and polish with 10X is cutting edge and is married with the use of the SALSA2 software to scaffold with Hi-C. The final assembly is well quality controlled with the use of an appropriate BUSCO database as well as BlobToolKit providing interesting information on the potential integration of microsporidian sequence into the genome.

 The genome note is of sufficient detail throughout, but I wonder if it would help reproducibility to include the specific settings used for the bioinformatic tools? 

 Analyses of datasets generated are presented clearly in large, well coloured figures with informative captions. There are also interactive versions of some figures available. The assembly data is accessible and linked in the manuscript.

Are sufficient details of methods and materials provided to allow replication by others?

Partly

Is the rationale for creating the dataset(s) clearly described?

Yes

Are the datasets clearly presented in a useable and accessible format?

Yes

Are the protocols appropriate and is the work technically sound?

Yes

Reviewer Expertise:

Evolutionary Genomics

I confirm that I have read this submission and believe that I have an appropriate level of expertise to confirm that it is of an acceptable scientific standard.
